# DNA Barcoding for Herbarium Specimens of the Red Alga *Meristotheca pilulaora* and Molecular Marker Development for Species Identification

**DOI:** 10.3390/biology15050424

**Published:** 2026-03-05

**Authors:** Soon Jeong Lee, Eun-Young Lee, Bo Yeon Kim, Sang-Rae Lee

**Affiliations:** 1 Tongyeong Regional Office, National Fishery Products Quality Management Service (NFQS), Tongyeong 53019, Republic of Korea; leesj73@korea.kr; 2 Biodiversity Research Department, Biodiversity Conservation Research Division, National Institute of Biological Resources, Incheon 22689, Republic of Korea; eylee1@korea.kr; 3 Seaweed Research Institute, National Institute of Fisheries Science, Haenam 59002, Republic of Korea; boyeon87@korea.kr; 4 Marine Research Institute, Pusan National University, Busan 46241, Republic of Korea

**Keywords:** *cox*1, *Gracilaria textorii*, *Meristotheca* *pilulaora*, molecular markers, *rbc*L, red algae

## Abstract

The genus *Meristotheca* (Gigartinales, Solieriaceae) comprises edible red algae that are economically important food ingredients in Korea, Japan, and China. In Korea, two species, *Meristotheca coacta* and *Meristotheca papulosa*, have been identified, with the latter being predominantly reported. Recently, molecular phylogenetic analysis enabled the identification of *Meristotheca pilulaora* (Gigartinales; Solieriaceae) on Jeju Island (Korea). In this study, we used a DNA barcoding method to re-examine *M. papulosa* herbarium specimens deposited at the National Institute of Biological Resources (Incheon, Korea). Specimens were collected from Korean coastal regions between 2009 and 2019. Molecular analyses based on the *rbc*L and *cox*1 sequences of the “*M. papulosa*” herbarium specimens revealed that the specimens were of two other species, *M. pilulaora* and *Gracilaria textorii* (Gracilariales; Gracilariaceae). Our work represents a case study for establishing a misidentification at the inter-ordinal level among herbarium specimens without DNA sequence verification. Moreover, we developed a molecular marker for the effective species-level identification of *M. pilulaora* and *G. textorii* specimens. The DNA barcoding method provides useful information regarding *M. pilulaora* distribution and taxonomy.

## Simple Summary

The red alga *Meristotheca pilulaora* (Gigartinales, Solieriaceae) has recently been described and newly recorded from Korea based on analyses of a species previously reported as *Meristotheca papulosa*. We re-examined *M. papulosa* herbarium specimens deposited in the National Institute of Biological Resources algal herbarium (Korea) to clarify the taxonomic relationship between *M. papulosa* and *M. pilulaora*. Using DNA barcoding, we examined *M. papulosa* specimens and unexpectedly identified *Meristotheca pilulaora* and *Gracilaria textorii* (Gracilariales, Gracilariaceae), but not *M. papulosa*. Thus, we revealed a misidentification at the inter-ordinal level (6/12 specimens). Moreover, we developed a *cox*1 marker as an effective molecular tool to discriminate between *M. pilulaora* and *G. textorii*. The molecular taxonomic results obtained in this study could contribute to gaining useful genetic information for re-examining herbarium specimen identities with similar morphologies and for establishing the geographic distribution of *M. pilulaora* in Korea.

## 1. Introduction

*Meristotheca* (Solieriaceae, Rhodophyta) species are edible red algae with economic value in Korea, Japan, and China [1,2]. The genus comprises species distributed worldwide [3]. To date, three *Meristotheca* species (*M. coacta*, *M. papulosa*, and *M. pilulaora*) have been reported from Korea [4]. *Meristotheca papulosa* has been widely reported in the Indo-Pacific region [3], whereas *M. coacta* has only rarely been included in the floristic list [4,5]. *Meristotheca pilulaora* was described as a new species, which was previously considered as *M. papulosa* on Jeju Island (Korea) [6].

The type locality of *M. papulosa* (Montagne) J. Agardh 1872 is Yemen, in the southern Arabian Peninsula and it has been reported from Africa, Asia, Australia, New Zealand, and the Pacific Islands [3]. Ecological and physiological studies on *M. papulosa* have been performed in Korea and Japan [2,7]. In Korea, *M. papulosa* has been reported only from Jeju Island [2,4,5,6].

DNA analyses of herbarium specimens could be used to explore the species diversity and identify new taxonomic entities, including novel and unrecorded species [8,9,10,11,12,13,14]. Taxonomists spend considerable time collecting specimens from various regions. Even though herbarium specimen analyses are likely to provide a diverse and unbiased approach to species classification, several biases could be introduced [10]. Therefore, morphological species identification without genetic information would likely establish the misidentification of related species with morphological similarities [11].

Numerous specimens are stored in herbaria worldwide. Therefore, DNA barcoding of herbarium specimens could be a useful taxonomic tool for identifying species. Moreover, the genetic diversity of the collected samples could provide new taxonomic information, including the identification of entirely new species and species previously unrecorded in local regions [8,9,10,11,12,13,14,15].

A recent rapid reduction in natural *M. papulosa* populations on Jeju Island in Korea indicates the necessity to monitor fluctuations in *Meristotheca* populations [2]. The National Institute of Biological Resources (NIBR, Korea; https://species.nibr.go.kr/index.do; accessed on 31 January 2026) possesses *M. papulosa* herbarium specimens, including those from a recent study on an algal herbarium, indicating broad distribution of the species around the Korean peninsula [5].

The verification of herbarium specimens would provide not only accurate taxonomic information based on morphological and genetic data, but also important basic information for future industrial use. The new genetic resources obtained from algal herbarium specimens could present important information about seaweeds that exist in natural ecosystems, as the specimens contain data such as the collection site and date of collection from natural seaweed beds [2,7].

In this study, we re-examined *M. papulosa* herbarium specimens collected from the Korean Peninsula and developed a molecular marker to discriminate species with similar morphologies. Identification of a given species is the first step in estimating the economic and ecological value of seaweeds. In particular, accurate identification of the taxonomic entities of *M. papulosa* specimens and the development of new molecular markers could provide useful information for further studies in taxonomy and aquaculture.

## 2. Materials and Methods

**Figure 1:**
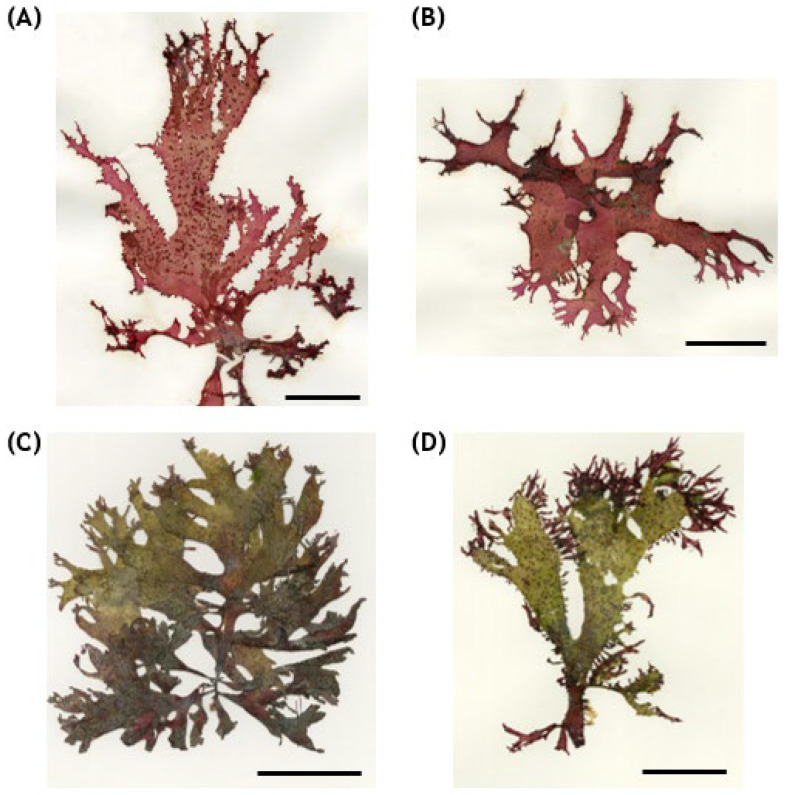
Morphological similarity between Korean *Meristotheca pilulaora* and *Gracilaria textorii* deposited in the algal herbarium of the National Institute of Biological Resources (NIBR). Those specimens were originally identified as *Meristotheca papulosa* and reidentified using DNA barcoding in this study. (*Meristotheca pilulaora* ((**A**)—NIBRAL0000147240 (Hado-ri, Jeju Island), (**B**)—NIBRAL0000138926 (Daejeong-eup, Jeju Island)); *G. textorii* ((**C**)—NIBRAL0000115411 (Geoje-si, Gyeongsangnam-do), (**D**)—NIBRAL0000147886 (Geoje-si, Gyeongsangnam-do)) (Scale bars = 5 cm).

In this study, herbarium specimens of *Meristotheca papulosa* from the NIBR (Korea) were analyzed (Table 1 and Table 2; Figure 1). We focused on the molecular analyses of specimens identified as *M. papulosa* from previous field surveys based on morphological examination.

Considering the collection sites and dates, we selected 22 sheets of *M. papulosa* herbarium specimens collected around the Korean Peninsula and successfully identified 12 specimens (Table 1 and Table 2; Figure 1). We obtained a small amount (<0.5 cm^2^) of sample from a single herbarium specimen for DNA extraction; the extracted DNA was subsequently subjected to polymerase chain reaction (PCR) analysis and sequencing as described previously [11]. Total genomic DNA was extracted using the DNeasy Plant Mini Kit (Qiagen, Germantown, MD, USA) following previously described protocols [11].

**Figure 2:**
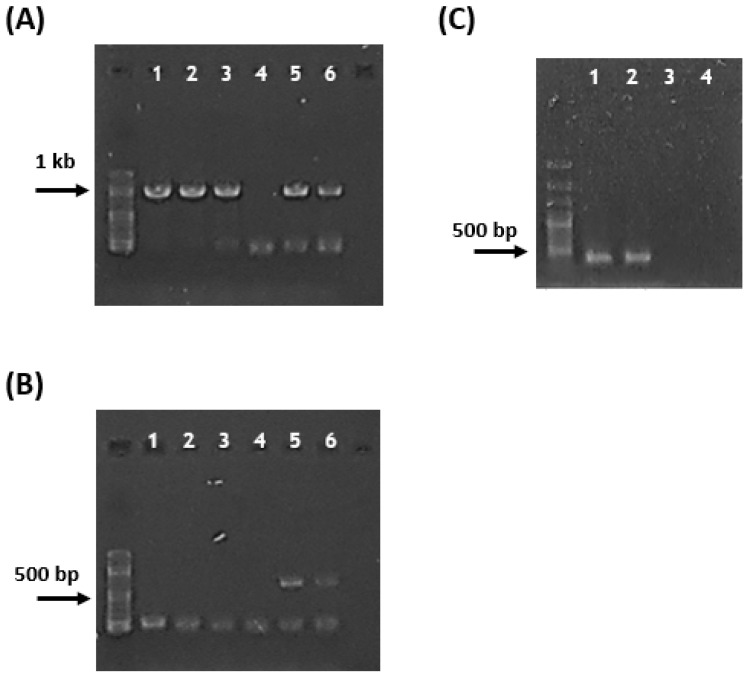
Amplification of *rbc*L and *cox*1 regions in Korean *Meristotheca pilulaora* and *Gracilaria textorii* herbarium specimens deposited in the algal herbarium of the National Institute of Biological Resources (NIBR) under *M. papulosa*. (**A**) Universal *rbc*L amplification for *Gracilaria* and *Meristotheca* species (*G. textorii*; line 1—NIBRAL0000147886, line 2—NIBRAL0000148336, line 3—NIBRAL0000148567) and *M. pilulaora*; line 5—NIBRAL0000154265, line 6—NIBRAL0000154300). (**B**) *Meristotheca pilulaora cox*1 amplification (line 5—NIBRAL0000154265, line 6—NIBRAL0000154300); no amplification for *G. textorii*. (**C**) *Gracilaria textorii cox*1 amplification (*G. textorii*; line 1—NIBRAL0000115705, line 2—NIBRAL0000143530); no amplification for *M. pilulaora* (line 3—NIBRAL0000154265, line 4—NIBRAL0000154300).

We used multiple molecular markers based on reference sequences deposited in GenBank (National Center for Biotechnology Information, NCBI). Two organelle-coding genes (*rbc*L, a plastid gene; *cox*1, a mitochondrial gene) were selected as molecular markers. New primers for the *rbc*L and *cox*1 regions were designed. Forward (rbcL-Red-8F; 5′-AATCTGTAGAAGAACGGACA-3′) and reverse (rbcL-Gig-978R; 5′-TACACCWGCCATACGCATCC-3′) primers were designed to amplify a 971-bp fragment in the *rbc*L region of the herbarium specimens (Figure 2A).

For the amplification of the *cox*1 region, two sets of *cox*1 primers were designed. For *Meristotheca* species, forward (cox1-Mer-84F; 5′-TGGTGCYTTTTCAGGTTTAATWGGA-3′) and reverse (cox1-Gig-749R; 5′-TCAGGGTGGCCRAARAAYCA-3′) primers were designed (Figure 2B) with 666-bp amplicons. For *G. textorii*, a different cox1-GraT-378F/cox1-Gig-749R primer set was used (cox1-GraT-378F (5′- CGAAGTTGGTGTAGGGACAGTA 3′)) with 372-bp amplicons (Figure 2C).

PCR was performed with an initial denaturation step at 94 °C for 3 min, followed by 40 cycles at 94 °C for 30 s, 50 °C for 30 s, and 72 °C for 1 min; and a final extension step at 72 °C for 7 min using Gene Amp-PCR-System-9700 (Applied Biosystems, Foster City, CA, USA). *AmfiXpand* PCR Master Mix (GenDEPOT, Katy, TX, USA) was used for PCR, and the products were sequenced using a commercial sequencing service provider (GenoTech, Daejeon, Republic of Korea). Chromatograms of sequencing were assembled in both directions using the Sequencher 5.4.6 software (Gene Codes, Ann Arbor, MI, USA). The sequencing quality was checked using the chromatograms, and we used the high-quality region in the sequencing data.

**Figure 3:**
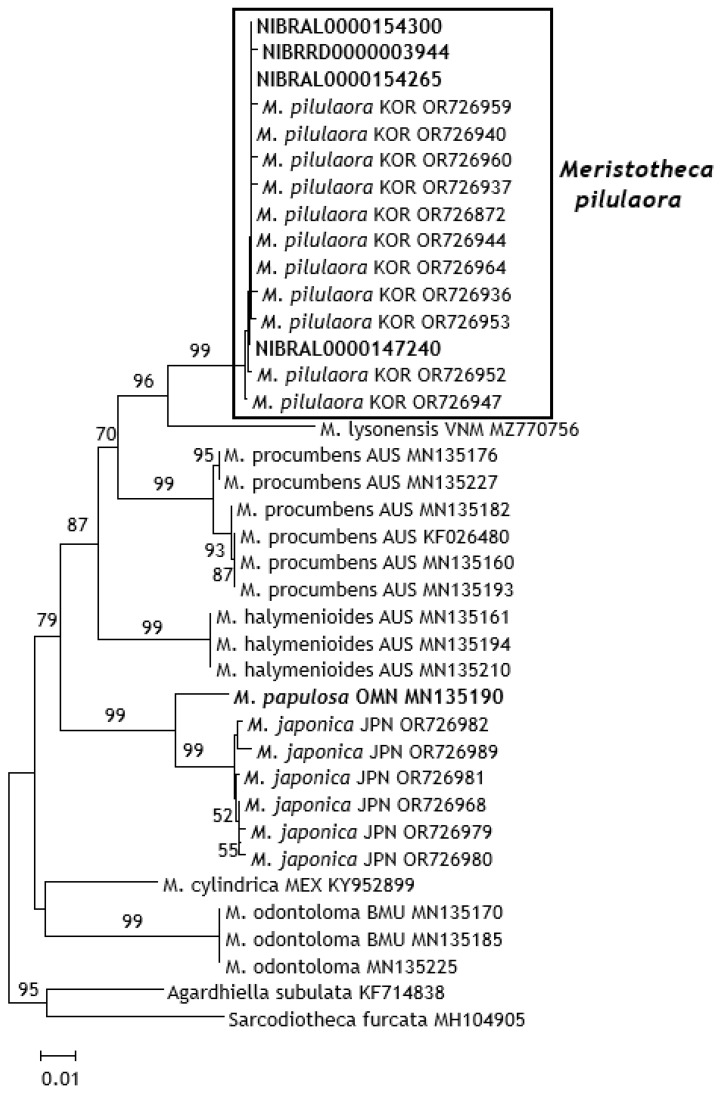
Phylogenetic tree of *cox*1 gene sequences constructed using neighbor-joining (Kimura 2-parameter model). *Agardhiella* and *Sarcodiotheca* species were selected as outgroups. Bootstrap values are presented on the branches (>50%).

We successfully isolated the *rbc*L and *cox*1 sequences from 12 herbarium specimens of *M. papulosa*. The DNA sequences have been deposited in GenBank (the accession numbers are presented in Table 1 and Table 2). DNA sequences (*rbc*L and *cox*1) isolated from Korean herbarium specimens were compared with reference sequences deposited in GenBank at the NCBI (*Meristotheca pilulaora* (*rbc*L and *cox*1) and *Gracilaria textorii* (ptDNA NC_046043 and mtDNA NC_037892) (Figure 3).

For molecular phylogenetic analyses, reference sequences for *cox*1 were obtained from GenBank (NCBI) (Figure 3). Molecular phylogenetic analyses were conducted using MEGA ver. 6 [15] using the neighbor-joining and maximum likelihood methods with 2000 bootstrap replicates. The pairwise distances were calculated using Kimura’s two-parameter method. Outgroups were selected based on a previous phylogenetic study of Solieriaceae [16].

## 3. Results

We obtained four *rbc*L gene sequences [OP554369–OP554371 (878 bp) and OP554372 (836 bp)] and four *cox*1 gene sequences (622 bp of OQ594746–OQ594749 (622 bp)] from six specimens (*Meristotheca pilulaora*; Table 1, Figure 2). We isolated six *rbc*L [PX613529–PX613534 (931 bp)] and six *cox*1 [PX613535—PX613540 (330 bp)] DNA sequences from six specimens and used them for species identification (*Gracilaria textorii*; Table 2, Figure 2). The primer pair of cox1 marker (cox1-Mer-84F/cox1-Gig-751R) produced 668-bp amplicons from *M. pilulaora* DNA templates, but not from *G. textorii* DNA samples (Figure 2B). The primer combination cox1-GraT-378F/cox1-Gig-751R of *cox*1 marker for *Gracilaria* species produced 374-bp amplicons from *G. textorii* DNA templates (Figure 2C).

Using BLAST search (GenBank, NCBI), we identified six “*M. papulosa*” specimens as *M. pilulaora*, recently established in Korea [6] (Table 1, Figure 3). Moreover, the other six “*M. papulosa*” specimens exhibited 100% identity with *G. textorii* (ptDNA NC_046043 and mtDNA NC_037892) (Table 2).

The *rbc*L sequences of five “*M. papulosa*” specimens contained the same DNA sequence and showed 100% identity with those of *M. pilulaora* populations. The *cox*1 gene of four “*M. papulosa*” specimens showed a difference of just one base among the specimens (NIBRRD0000003944 differed by one base from the sequences of the other specimens), demonstrating 99.8–100% identity with that of the *M. pilulaora* population in GenBank. Moreover, the “*M. papulosa*” specimens showed 93.7–89.8% *cox*1 sequence variation at the interspecific level. Therefore, the “*M. papulosa*” specimens examined in this study could be re-identified as *M. pilulaora*, as reported previously [6] (Figure 3).

In this study, we revealed a high level of misidentification (i.e., 50%, corresponding to 6 out of 12 specimens) of *G. textorii*, which was originally identified as *M. papulosa*. Moreover, we developed molecular markers for effective identification of *M. pilulaora* and *G. textorii* herbarium specimens. Moreover, *M. pilulaora* and *G. textorii* were successfully distinguished by the electrophoresis experiments using *cox*1 molecular markers (Figure 2B,C).

## 4. Discussion

The herbarium DNA barcoding has enabled to identify the biodiversity [8,9,10,11,12,13,14,15]. During the taxonomic re-examination of herbarium specimens of *M. papulosa* in the NIBR, we identified new genetic and species diversity in the algal herbarium (e.g., [11]).

Six of the 12 “*M. papulosa*” specimens examined were identified as *M. pilulaora* (Gigartinales; Solieriaceae) and the other six as *G. textorii* (Gracilariales; Gracilariaceae). Although *M. papulosa* and *M. coacta* have been reported by previous studies on Korean algal flora [4,5], they were not identified in this study. In a previous study [6], *M. pilulaora* was found only in the coastal regions of Jeju Island (Korea). In contrast, the herbarium DNA barcoding method used in the present study revealed the distribution of *M. pilulaora*, for the first time, on Ulleung Island, Korea (Table 1).

Regarding the Korean species of the genus *Gracilaria*, molecular phylogenetic studies were conducted with morphological observations [17,18]. We uncovered a misidentification at the inter-ordinal level between *M. pilulaora* and *G. textorii* (6/12 samples) (Table 2). Thus, most herbarium specimens lacking evidence of DNA sequences could have been taxonomically misidentified (e.g., [11]). Therefore, taxonomic re-examination is required to verify herbarium specimens. Our approach can provide a case study for the DNA barcoding method for herbarium specimens, and the molecular markers developed in this study represented an effective molecular tool for the species-level identification between *M. pilulaora* and *G. textorii*.

## 5. Conclusions

Many taxonomists worldwide have spent considerable time searching for new and unrecorded species. Moreover, numerous herbarium specimens have been deposited without DNA evidence to confirm species identification. Our work represents a case study for establishing misidentification in herbarium specimens and finding a new geographic distribution. Therefore, the DNA barcoding analysis for herbarium specimens can provide the useful information for algal taxonomic studies.

## Figures and Tables

**Table 1:** Revised *Meristotheca pilulaora* specimens previously listed under *Meristotheca papulosa*.

Collection Site	Herbarium Number	Collection Date	*rbc*L	*cox*1
Cheonbu-ri, Ulleung Island	NIBRAL0000154265	19 May 2015	OP554370	OQ594746
Jeodong-ri, Ulleung Island	NIBRAL0000154300	20 May 2015	OP554369	OQ594747
Hallim-eup, Jeju Island	NIBRRD0000003944	5 May 2019	OP554371	OQ594748
Daejeong-eup, Jeju Island	NIBRAL0000138926	30 May 2013	OP554371	-
Hado-ri, Jeju Island	NIBRAL0000147240	11 August 2014	-	OQ594749
Munseom, Jeju Island	NIBRRD0000005973	26 July 2010	OP554372	-

**Table 2:** Revised *Gracilaria textorii* specimens previously listed under *Meristotheca papulosa*.

Collection Site	Herbarium Number	Collection Date	*rbc*L	*cox*1
Geoje-si, Gyeongsangnam-do	NIBRAL0000115411	23 June 2009	PX613529	PX613535
Geoje-si, Gyeongsangnam-do	NIBRAL0000115705	24 June 2009	PX613530	PX613536
Namhae-gun, Gyeongsangnam-do	NIBRAL0000143530	4 July 2013	PX613531	PX613537
Geoje-si, Gyeongsangnam-do	NIBRAL0000147886	12 July 2014	PX613532	PX613538
Jindo-gun, Jeollanam-do	NIBRAL0000148336	28 July 2014	PX613533	PX613539
Wando-gun, Jeollanam-do	NIBRAL0000148567	26 September 2014	PX613534	PX613540

## Data Availability

The sequence data have been deposited in GenBank under accession numbers (https://www.ncbi.nlm.nih.gov).

## Abbreviations

The following abbreviations are used in this manuscript:

NCBI	National Center for Biotechnology Information
NIBR	National Institute of Biological Resources
PCR	Polymerase chain reaction
